# Differential impacts of ambient PM2.5 exposure on sperm quality in northern Thailand

**DOI:** 10.5935/1518-0557.20240051

**Published:** 2024

**Authors:** Aram Thapsamuthdechakorn, Tawiwan Pantasri, Usanee Sanmee, Tanarat Muangmool, Pareeya Somsak, Pannarai Somboonchai, Jamjit Doungpunta

**Affiliations:** 1 Department of Obstetrics and Gynecology, Faculty of Medicine, Chiang Mai University, Chiang Mai, Thailand; 2 Fertility Center, Center of Medical Excellence Clinic, Faculty of Medicine, Chiang Mai University, Chiang Mai, Thailand

**Keywords:** air pollution, PM 2.5, sperm quality, semen analysis

## Abstract

**Objective:**

This study aimed to explore the correlation between ambient particulate matter 2.5 (PM2.5) concentration and sperm quality among northern Thai men exposed to the seasonal air pollution from the agricultural burning process.

**Methods:**

The demographic data and semen analysis of Thai men living in Chiang Mai, Thailand, who visited the infertile clinic were collected. The correlation test between the monthly amount of PM2.5 and sperm quality was carried out.

**Results:**

From 2017 to 2021, 1,109 Thai men visited the Infertile Clinic. The correlation test between PM2.5 and sperm quality in years with a better climate revealed a weak positive correlation between the mean PM2.5 and percentage of progressive motile sperm and normal morphology (r=0.08, *p*=0.05 and r=0.1, *p*=0.02). However, there was a negative correlation between the mean PM2.5 and sperm concentration, progressive motility and normal sperm morphology during the years with a higher amount of ambient PM2.5, and especially PM2.5 exposure 3 months before semen collection (r=-0.12, *p*=0.01, r=-0.11, *p*=0.003, r=-0.15, *p*=0.004).

**Conclusions:**

Exposure to a high amount of PM2.5 air pollution negatively affects sperm quality.

## INTRODUCTION

Air pollution has a detrimental effect on health (Pothirat *et al.*, 2019). There are various pollutants in the air including PM10, PM2.5, nitrogen dioxide, and ozone (Pothirat *et al.*, 2019). Ambient PM2.5 (particulate matter smaller or equal to 2.5 micrometer; PM 2.5) is associated with respiratory problems and cardiovascular disease (Pothirat *et al.*, 2019; Nakharutai *et al.*, 2022). Exposure to ambient PM2.5 and nitrogen dioxide has the highest impact on all-cause mortality in northern Thailand (Pinichka *et al*. 2017).

PM2.5 inhalation directly affects alveolar cells, increases oxidative stress and causes pulmonary injury (Saffari *et al.*, 2014; Snow *et al.*, 2014; Liu *et al.*, 2020). It also relates to systemic inflammation (Chin, 2015). Furthermore, PM2.5 exposure causes testicular injury and germ cell apoptosis in rats (Liu *et al.*, 2017).

Studies regarding air pollution affecting sperm quality encompasses various reports that ranged from both high to low impact. The various constituents of air pollutants; amount and duration of exposure, might explain this contradiction (Hansen *et al*., 2010; Wu *et al*., 2017; Wu *et al*., 2021; Lao *et al*., 2018; Nobles *et al*., 2018; Chansuebsri *et al*., 2022). Strong correlations of PM2.5 exposure, 2-3 months before the time of semen analysis, gave hits that such exposure could play a role in the detrimental effect on spermatogenesis (Hammoud *et al.*, 2010; Wu *et al.*, 2017).

Chiang Mai is a northern province of Thailand, located in a basin-like terrain surrounded by mountains. People in the rural area mostly work in agriculture. The dry season is the smoky haze pollution period in Chiang Mai (Thepnuan *et al.*, 2020). During this highly intense smoky period, the source of PM2.5 is a mixture of burning biomass, traffic exhaust fumes and transboundary pollution (Thepnuan *et al.*, 2020; Chansuebsri *et al.*, 2022).

There are few reports about male fertility being affected by seasonal PM2.5 from the agricultural process. Therefore, the aim of this study was to explore the effect of ambient PM2.5 on male fertility.

## METHODS

Data were collected from the medical records of men living in northern Thailand who underwent semen analysis at the Infertile Clinic, Chiang Mai University between January 2017 and December 2021. The data included demographics, sperm parameters and outcome of fertility treatment.

The semen analysis was carried out using an HTM IVOS II computer assisted semen analysis (CASA; Hamilton Throne Biosciences, Beverly, MA), equipped with Clinical Human Motility II Software to determine: sperm concentration, progressive motility and morphology.

Monthly data of Chiang Mai ambient PM2.5 concentration, between January 2017 and December 2021, were collected from the Thai Pollution Control Department. Both the mean and maximum value of ambient PM2.5 were reported during the study period. A level of ambient PM2.5 over 50 µg/m^3^ was considered unhealthy.

This study was approved by the Ethical Committee of the Faculty of Medicine, Chiang Mai University with the exemption of the informed consent (OBG-2564-08563).

In order to compare the difference between two groups of data, the t-test was applied for continuous data, with normal distribution. Otherwise, the non-parametric test was used. The comparison between various groups of continuous data was carried out by the ANOVA test. The difference of categorical datasets was determined by the Chi-squared test. The relationship between ambient PM2.5 concentration and sperm parameters were analyzed by Pearson’s correlation coefficients. Univariable and multivariable logistic regression was conducted to identify the potential factors associated with the chemical pregnancy rate.

All statistical tests were performed with the statistical package for social science (SPSS, USA version 22.0). A *p*-value of less than 0.05 was considered statistically significant.

## RESULTS

During the study period, there was a high level of PM2.5 in a particular period of each year. There was a difference of ambient PM2.5 concentration in these five years (2017 to 2021). The mean ambient PM2.5 was highest in 2019 and lowest in 2017. The maximum ambient PM2.5 was highest in 2019 and lowest in 2020, as shown in Table 1a.

**Table 1a:** Average of mean and maximum ambient PM2.5 concentration between 2017 and 2021.

Year	Mean PM2.5 (± SD) (µg/m^3^)	Mean of maximum PM 2.5 (± SD) (µg/m^3^)	*p*-value
2017	23.3±13.8	39.8±25.3	<0.001^*^
2018	27.0±18.2	46.5±26.4	
2019	32.0±23.2	62.6±54.3	
2020	22.4±19.7	41.0±37.9	
2021	28.9±24.9	49.6±41.2	
**Overall**	48.1±39.0	48.1 ± 39.0	

* *p*<0.001 both mean and maximum value.

According to the difference in levels of ambient PM2.5 in individual years, the data was broken down into two groups. Group 1 had the data from 2017, 2018 and 2020 and Group 2 the years with higher ambient PM2.5, which were 2019 and 2021 (Table 1b).

**Table 1b:** Comparison of ambient PM2.5 concentration between Group 1 (2017, 2018 and 2020) and 2 (2019 and 2021).

Ambient PM2.5 concentration (µg/m^3^)	Group 1 (2017,2018,2020)	Group 2 (2019,2021)	*p*-value
Maximum value	42.5±29.4	56.9±49.4	<0.001^*^
Mean value	24.5±17.2	30.7±24.0	<0.001^*^

The participants comprised 1,109 men attending the Infertile Clinic for semen analysis during the 5-year study period (2017 to 2021). The majority of them were reported as healthy and were involved in fertility management. The mean age was 34.6 years and the mean body mass index was 25.2 kg/m^2^. The demographic data of men undergoing semen analysis in Groups 1 and 2 were not statistically significant, as shown in Table 2.

**Table 2:** Characteristics of the study subjects in Group 1 (2017, 2018 and 2020) and 2 (2019 and 2021).

Characteristics	Group 1 (n=678)	Group 2 (n=431)	*p*-value
Age (Years) <25 >25 Mean (±SD)	32 (4.7) 646 (95.3) 34.6±7.0	11 (2.6) 420 (97.4) 34.6±6.2	0.07
Body Mass Index (kg/m^2^) (n=1,064) <18.5 18.5-24.9 25-29.9 >30 Mean (± SD)	16 (2.5) 334 (51.8) 230 (35.7) 65 (10.1) 25.0±3.	7 (1.7) 205 (48.9) 158 (37.7) 49 (11.7) 25.5±4.1	0.55
Underlying disease (n=995) No Yes	484 (85.7) 81 (14.3)	370 (86.0) 60 (14.0)	0.86
Smoking status (n=1,108) Non-smoker Smoker	549 (81.1) 128 (18.9)	342 (79.4) 89 (20.6)	0.48
Infertility factor of couples (n=979) Male factor infertility Tubal factor Ovulation factor Endometriosis Unexplained Other	248 (41.8) 50 (8.4) 60 (10.1) 58 (9.8) 137 (23.1) 41 (6.9)	138 (35.8) 38 (9.9) 50 (13.0) 28 (7.3) 108 (28.1) 23 (6.0)	0.13

**Figure 1:**
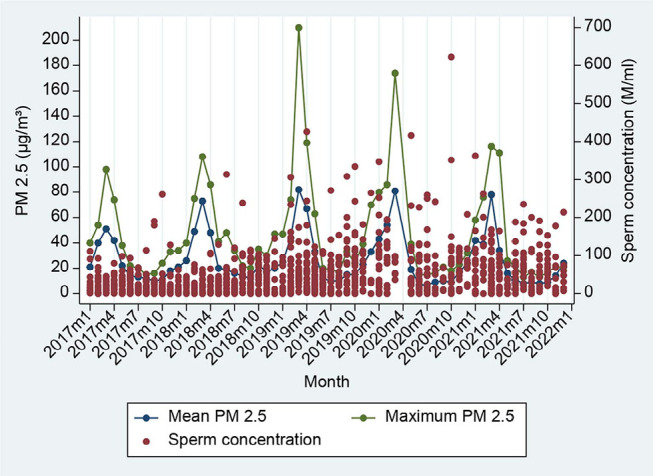
Ambient PM2.5 in each month and pool data of sperm concentration.

**Figure 2:**
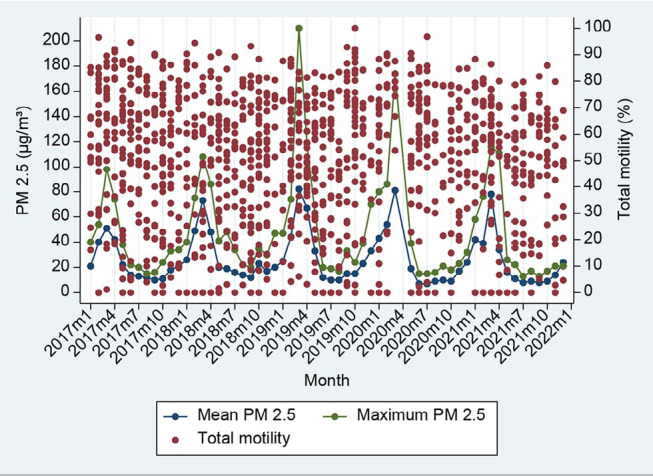
Ambient PM2.5 in each month and pool data of total sperm motility percentage.

**Figure 3:**
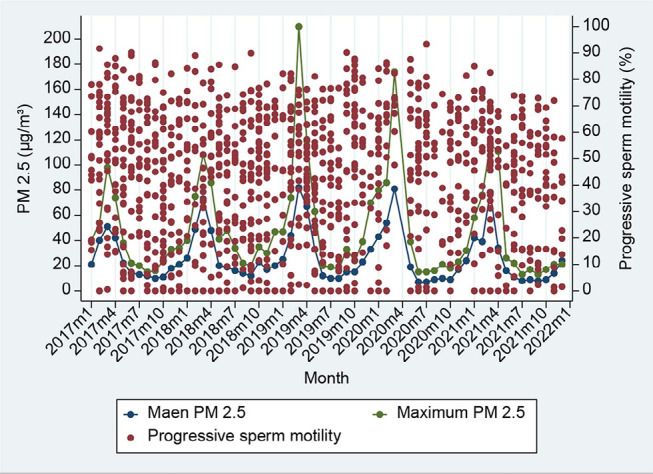
Ambient PM2.5 in each month and pool data of progressive sperm motility percentage.

**Figure 4:**
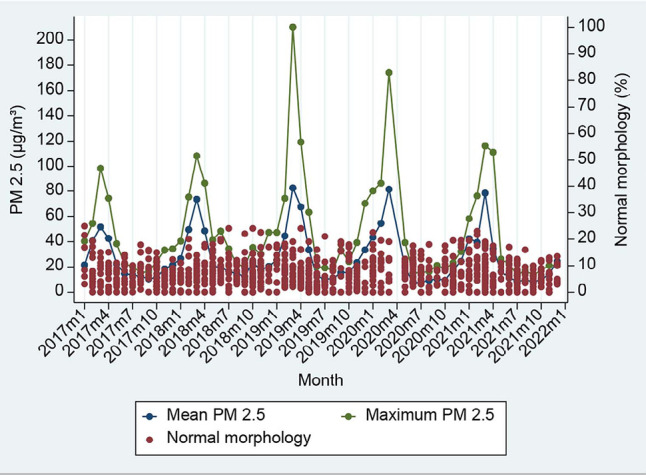
Ambient PM2.5 in each month and pool data of normal sperm morphology percentage.

Data on homogenous sperm parameters were available during the study period, while PM2.5 values had seasonal variation, as shown in Figures 1-4.

Overall data revealed weak positive correlation of the progressive sperm motility percentage and normal morphology at the time of PM2.5 exposure, as shown in Table 3.

**Table 3:** Correlation of monthly mean ambient PM2.5 (µg/m^3^) concentration between 2017 and 2022 and sperm parameters in different periods of exposure.

Sperm parameters	PM 2.5 exposure (mean value)	Coefficients (95%CI)	*p*
Concentration (M/ml)	On time	0.061 (-0.105 - 0.228)	0.47
	1-month lag	0.005 (-0.156 - 0.166)	0.95
	2-months lag	-0.108 (-0.262 - 0.045)	0.17
	3-months lag	-0.079 (-0.223 - 0.066)	0.29
Total motile sperm (%)	On time	0.069 (-0.005 - 0.142)	0.07
	1-month lag	0.016 (-0.061 - 0.092)	0.69
	2-months lag	-0.026 (-0.098 - 0.047)	0.49
	3-months lag	-0.036 (-0.105 - 0.033)	0.31
Progressive motility (%)	On time	0.091 (0.018 - 0.164)	0.015^*^
	1-month lag	0.059 (-0.017 - 0.136)	0.13
	2-months lag	0.016 (-0.056 - 0.088)	0.66
	3-months lag	-0.012 (-0.080 - 0.056)	0.73
Normal morphology (%)	On time	0.020 (0.003 - 0.037)	0.021^*^
	1-month lag	0.016 (-0.002 - 0.034)	0.09
	2-months lag	0.004 (-0.013 - 0.020)	0.65
	3-months lag	-0.005 (-0.020 - 0.010)	0.53

# 1-month lag: semen analysis 1 month following PM2.5 exposure

2-months lag: semen analysis 2 months following PM2.5 exposure

3-months lag: semen analysis 3 months following PM2.5 exposure

Subgroup analysis of the correlation between ambient PM2.5 and sperm parameters in years with better climate revealed weak positive correlation of progressive motile sperm percentage following 1 month of PM2.5 exposure and 2 months of normal morphologic sperm percentage (r=0.08, *p*=0.05 and r=0.1, *p*=0.02), as shown in Table 4. However, there was a negative correlation between each sperm parameter and level of PM2.5, especially following 3 months exposure before semen analysis in the year with worse climate (r=-0.12, *p*=0.01, r=-0.11, *p*=0.003, r=-0.15, *p*=0.004), as shown in Table 5.

**Table 4:** Correlation of monthly mean ambient PM2.5 (µg/m^3^) concentration between 2017, 2018 and 2020 and sperm parameters in different periods of exposure (Group 1; n=678).

Sperm parameters	PM2.5 exposure (Mean value )	Correlation coefficient	p-value
Concentration (M/ml)	On time 1-month lag 2-months lag 3-months lag	-0.029	0.45
		-0.026	0.51
		0.013	0.74
		0.029	0.45
Total motile sperm (%)	On time 1-month lag 2-months lag 3-months lag	0.037	0.34
		0.038	0.33
		0.036	0.36
		0.045	0.24
Progressive motility (%)	On time 1-month lag 2-months lag 3-months lag	0.060	0.12
		0.076	0.049^*^
		0.069	0.07
		0.060	0.12
Normal morphology (%)	On time 1-month ag 2-months lag 3-months lag	0.018	0.68
		0.064	0.14
		0.100	0.02^*^
		0.083	0.06

# 1-month lag : semen analysis 1 month following PM2.5 exposure

2-months lag : semen analysis 2 months following PM2.5 exposure

3-months lag : semen analysis 3 months following PM2.5 exposure

**Table 5:** Correlation of monthly mean ambient PM 2.5 (µg/m^3^) concentration between 2019 and 2021 and sperm parameters in different periods of exposure (Group 2; n=431).

Sperm parameters	PM2.5 exposure (Mean value )	Correlation coefficient	*p*-value
Concentration (M/ml)	On time	0.018	0.72
	1-month lag	-0.012	0.80
	2-months lag	-0.122	0.011^*^
	3-months lag	-0.122	0.012^*^
Total motile sperm (%)	On time	0.098	0.043^*^
	1-month lag	-0.009	0.86
	2-months lag	-0.100	0.038^*^
	3-months lag	-0.142	0.003^*^
Progressive motility (%)	On time	0.107	0.026^*^
	1-month lag	0.019	0.7
	2-months lag	-0.068	0.16
	3-months lag	-0.113	0.003^*^
Normal morphology (%)	On time	0.155	0.002^*^
	1-month lag	0.059	0.25
	2-months lag	-0.088	0.08
	3-months lag	-0.148	0.004^*^

# 1-month lag : semen analysis 1 month following PM2.5 exposure

2-months lag : semen analysis 2 months following PM2.5 exposure

3-months lag : semen analysis 3 months following PM2.5 exposure

Eighty-six men underwent fertility treatment within 1 month after semen analysis. The univariable analysis among these men revealed that PM2.5 exposure did not affect the chance of conception. The infertility treatment was the only factor affecting chemical pregnancy rate, as shown in Table 6.

**Table 6:** Associated factors of conception among men who received fertility treatment within 1 month after semen analysis (n=86).

Factors	Treatment Outcomes		Univariable analysis		Multivariable analysis	
	No pregnancy	Pregnancy	OR (95%CI)	*p*	aOR (95%CI)	*p*
Mode of treatment IUI IVF and ICSI	43 (93.5) 27 (67.5)	3 (6.5) 13 (32.5)	1 6.90 (1.80-26.47)	0.005^*^		
Infertility causes Male factor Female factor	31 (86.1) 39 (78.0)	5 (13.9) 11 (22.0)	1.75 (0.55-5.56)	0.344		
Year of research Group 1 Group 2	43 (81.1) 27 (81.8)	10 (18.9) 6 (18.2)	1 0.96 (0.31-2.93)	0.937		
PM2.5 on time (µg/m^3^) <50 ≥50	52 (82.5) 18 (78.3)	11 (17.5) 5 (21.7)	1.31 (0.40-4.30)	0.652	0.86 (0.23-3.26)	0.820
1-month lag (µg/m^3^) <50 ≥50	50 (83.3) 20 (76.9)	10 (16.7) 6 (23.1)	1.50 (0.48-4.68)	0.485	1.59 (0.45-5.63)	0.469
2-months lag PM2.5 (µg/m^3^) <50 ≥50	44 (86.3) 26 (74.3)	7 (13.7) 9 (25.7)	2.18 (0.72-6.54)	0.166	2.83 (0.80-10.04)	0.107
3-months lag PM2.5 (µg/m^3^) <50 ≥50	47 (83.9) 23 (76.7)	9 (16.1) 7 (23.3)	1.59 (0.53-4.81)	0.412	2.54 (0.69-9.32)	0.159

#Multivariable analysis was adjusted by the mode of treatment and cause of infertility,

aOR; adjusted Odds Ratio

IUI : Intrauterine insemination

IVF: in vitro fertilization

ICSI: intracytoplasmic sperm injection

## DISCUSSION

Air pollution is an important global issue. The extent of the problem is reported differently by countries, periods, years and various aspects of volatile toxins and a variety of particular matter (Wu *et al.*, 2017; Qi *et al.*, 2020; Franzin *et al.*, 2021; Chansuebsri *et al.*, 2022; Kahraman & Sivri, 2022; Pereira Barboza *et al.*, 2023)

Air pollution affects health by both direct inhalation and indirect pathways. The indirect effect from air pollution on male fertility is seen clearly in rat studies (Liu *et al.*, 2017; Liu *et al*., 2019). The pathogenesis would be systemic inflammation and oxidative stress (Chin, 2015; Liu *et al.*, 2017; Liu *et al*., 2019).However, there is still no clear explanation among human data.

The effect of ambient PM2.5 on sperm quality in this study is quite moderate. PM2.5 exposure for 2-3 months before semen analysis affects sperm quality more than exposure on time. It seems that PM2.5 affects the spermatogenesis process, which takes 70-90 days (Hammoud *et al.*, 2010; Wu *et al.*, 2017). Moderate results are similar to those in studies when ambient PM2.5 was not extremely high (Hansen *et al*., 2010; Lao *et al*., 2018; Nobles *et al*., 2018). The constituents of air pollution could be another important factor. The PM2.5 caused by industrial and traffic fumes contains more toxin and might have more detrimental effect on health and sperm quality (Hammoud *et al.*, 2010; Wu *et al.*, 2017; Wu *et al*., 2021; Chansuebsri *et al.*, 2022). The seasonal smoke haze from burning might be less harmful in that cell and tissue injury could have more time to self-repair.

A year with better climate and low level of ambient PM2.5, had a weak positive correlation between sperm quality and level of ambient PM2.5. This is contrary to the hypothesis of adverse effect on health from PM2.5. The result of weak positive correlation also was found in other studies (Hansen *et al.*, 2010; Lao *et al.*, 2018), where the level of ambient PM2.5 was not extremely high. It was hypothesized that the compensatory process might occur before the worst outcome, when chronic exposure or higher levels of PM2.5 appeared (Lao *et al.*, 2018). Further studies are necessary to confirm this hypothesis.

The outcome of fertility treatment among this small subgroup, within 1 month after semen analysis, revealed that the level of PM2.5 exposure did not impact the chance of conception. Nevertheless, a larger sample size is needed to conclude this hypothesis. The different types and levels of air pollution might have different effects on fertility outcome.

Air pollution has various aspects of volatile toxins and varied particular matter (Wu *et al.*, 2017; Chansuebsri *et al.*, 2022). This study explored only PM2.5, and no other aspects of air pollution. Other confounding factors affecting sperm quality could be temperature and humidity, which were not included in this study. The subjects in this study were rather homogenous in demography, which might not answer similar questions in other groups of the population. This study did not have data on the protection and habits among individuals regarding air pollution, such as outdoor activity, air filtering or filtered masks (Allen & Barn, 2020). Further study on oxidative stress and DNA fragmentation would fill the knowledge gap between air pollution and sperm quality.

In conclusion, exposure to a high levels of PM2.5 affects sperm quality negatively in Chiang Mai males. More studies are needed to explain the pathogenesis of PM2.5 affecting spermatogenesis.
